# How the Neurotoxin β-*N*-Methylamino-l-Alanine Accumulates in Bivalves: Distribution of the Different Accumulation Fractions among Organs

**DOI:** 10.3390/toxins12020061

**Published:** 2020-01-21

**Authors:** Alexandra Lepoutre, Elisabeth J. Faassen, A. J. Zweers, Miquel Lürling, Alain Geffard, Emilie Lance

**Affiliations:** 1 UMR-I 02 INERIS-URCA-ULH SEBIO, Unité Stress Environnementaux et BIOsurveillance des milieux aquatiques, UFR Sciences Exactes et Naturelles, Moulin de la Housse, BP 1039, 51687 Reims CEDEX 2, France; alexandra.lepoutre@univ-reims.fr (A.L.); alain.geffard@univ-reims.fr (A.G.); 2 Wageningen Food Safety Research, Wageningen Research, Akkermaalsbos 2, 6708 WB Wageningen, The Netherlands; els.faassen@wur.nl; 3 Aquatic Ecology and Water Quality Management Group, Wageningen University, Droevendaalsesteeg 3a, 6708 PB Wageningen, The Netherlands; miquel.lurling@wur.nl; 4 Department of Microbial Ecology, Netherlands Institute of Ecology (NIOO-KNAW), Droevendaalsesteeg 10, 6708 PB Wageningen, The Netherlands; H.Zweers@nioo.knaw.nl; 5 Equipe Cyanobactéries, Cyanotoxines et Environnement, UMR Molécules de Communication et Adaptation des Microorganismes (MCAM), Museum National Histoire Naturelle, CNRS, 12 rue Buffon, CP 39, 75231 Paris CEDEX 05, France

**Keywords:** β-Methylamino-l-alanine, organotropism, *Dreissena polymorpha*, BMAA

## Abstract

The environmental neurotoxin β-methylamino-l-alanine (BMAA) may represent a risk for human health. BMAA accumulates in freshwater and marine organisms consumed by humans. However, few data are available about the kinetics of BMAA accumulation and detoxification in exposed organisms, as well as the organ distribution and the fractions in which BMAA is present in tissues (free, soluble bound or precipitated bound cellular fractions). Here, we exposed the bivalve mussel *Dreissena polymorpha* to 7.5 µg of dissolved BMAA/mussel/3 days for 21 days, followed by 21 days of depuration in clear water. At 1, 3, 8, 14 and 21 days of exposure and depuration, the hemolymph and organs (digestive gland, the gills, the mantle, the gonad and muscles/foot) were sampled. Total BMAA as well as free BMAA, soluble bound and precipitated bound BMAA were quantified by tandem mass spectrometry. Free and soluble bound BMAA spread throughout all tissues from the first day of exposure to the last day of depuration, without a specific target organ. However, precipitated bound BMAA was detected only in muscles and foot from the last day of exposure to day 8 of depuration, at a lower concentration compared to free and soluble bound BMAA. In soft tissues (digestive gland, gonad, gills, mantle and muscles/foot), BMAA mostly accumulated as a free molecule and in the soluble bound fraction, with variations occurring between the two fractions among tissues and over time. The results suggest that the assessment of bivalve contamination by BMAA may require the quantification of total BMAA in whole individuals when possible.

## 1. Introduction

The environmental toxin β-Methylamino-l-alanine (BMAA) is a non-protein amino acid that may be involved in the development of neuro-degenerative pathologies such as the ALS-PDC syndrome (amyotrophic lateral sclerosis-parkinsonism-dementia) [1,2,3]. This environmental toxin is able to induce: (i) excitotoxicity by interacting with glutamate receptors in presence of bicarbonate at physiological concentrations, (ii) a dysregulation of the cellular protein homeostasis, and (iii) an inhibition of the cysteine/glutamate antiporter, leading to a potential oxidative stress [4]. BMAA could be misincorporated in human cells instead of the amino acid serine [5], which may lead to protein dysfunction, although this hypothesis has been criticized [6]. Moreover, BMAA interacts with neuromelanin into the central nervous system, which could lead to long-lasting neurotoxic activity [7].

The few existing data concerning potential BMAA producers show that some cyanobacteria (*Nostoc* sp., *Leptolyngbya* sp.), diatoms (*Chaetoceros* sp., *Phaeodactylum tricornutum*) and dinoflagellates (*Heterocapsa triquetra*, *Gymnodinium catenatum*) may produce it [8,9,10,11,12]. To date, no large screening of several cyanobacterial genus has been realized using a selective method of detection and quantification such as liquid chromatography coupled to tandem mass spectrometry (LC-MS/MS). Indeed, BMAA identification and quantification is the subject of an analytical controversy, and only the use of LC-MS/MS, with or without previous derivatization, appears appropriate [13,14]. In aquatic ecosystems worldwide, BMAA accumulates in primary consumers (bivalves and crustaceans) [13], and in secondary consumers as sharks [15]. The neurotoxin may also be biomagnified in marine [16] and continental food webs [17]. Human intoxication is thought to occur through a chronic exposure while ingesting BMAA-containing food [18]. However, little reliable data are available regarding the BMAA concentration in fresh waters as an intracellular form (in the phytoplankton biomass) or as a dissolved form [13]. Due to the analytical challenge in quantifying dissolved BMAA, the neurotoxin is commonly analyzed in the phytoplankton biomass with concentrations reaching 968 µg/g DW [14]. However, most reports regarding the presence of BMAA in cyanobacteria are negative [11,19,20].

Monitoring of substances in freshwater ecosystems can be facilitated using organisms as bioindicators. There are several requirements for using a species in biomonitoring: the species must be widespread, with a limited mobility, easy to handle and in direct contact with the substance in the medium [21,22,23]. *Dreissena polymorpha* is a freshwater filter-feeder bivalve that may be in direct contact with environmental toxins present in the water column like BMAA and its producers. Studies on *D. polymorpha* clearance rates and stomach contents showed that this species is able to ingest phytoplankton species like cyanobacteria and diatoms [24,25]. This species has been used as a sentinel organism of water quality in the Great Lakes from mid 1970s in the “Mussel watch” program [26] to monitor levels of bioavailable pollutants [27]. Moreover, *D. polymorpha* is able to accumulate BMAA during a short time (up to 48 h) exposure to radiolabelled BMAA [28]. In that study, the only fractions analyzed were free BMAA and precipitated bound BMAA. Indeed, BMAA may accumulate in free form (“free BMAA”) in tissues when extracted with polar solvents. BMAA may also be associated to unknown compounds that can stay in solution after extraction and precipitation, underlying a low molecular weight of the BMAA-molecule complex. This fraction is called “soluble bound BMAA”. BMAA can also be found bound in the precipitate, suggesting a heavier weight of the molecular complex. This fraction is called “precipitated bound BMAA”. However, there no data yet on the precursors of these fractions [29], although some authors suggested that BMAA binding to molecules requires the involvement of biological processes [30], each fraction requiring a different extraction procedure [29]. In addition, the total BMAA encompassing the free, soluble bound and precipitated bound fractions, can be directly quantified by an extraction with HCl [31]. 

The evaluation of BMAA occurrence in aquatic environments using integrator organisms requires a knowledge of the BMAA accumulation fractions that have to be analyzed, as well as an understanding of the kinetics of accumulation and detoxification and the distribution among tissues [32]. The present study aims to give a better understanding of total, free, soluble bound and precipitated bound BMAA accumulation and detoxification within organs of the freshwater mussel *D. polymorpha*, during a discontinuous exposure to 7.5 µg of dissolved BMAA/mussel/3 days for 21 days, followed by 21 days of depuration in clear water. Dissolved BMAA was chosen over the use of BMAA-producing organisms in relation to the absence of a known organism steadily producing BMAA over time [13]. Indeed, the few studies available to date show that in situ environmental factors or culture conditions such as nitrogen availability can influence BMAA production [33,34]. In addition, there is evidence that *D. polymorpha* can modify water biological and physicochemical characteristics by excreting ammoniums and dissolved phosphorus or depleting dissolved oxygen concentrations [35,36,37]. Therefore, the addition of mussels in the medium could potentially modulate BMAA production over time. The results are discussed in terms of the appropriate methodology, i.e. which BMAA accumulation fraction to quantify and which mussel organ to sample, which could be applied in the context of using *D. polymorpha* as a bioindicator of the presence of BMAA in fresh waters.

## 2. Results

**Figure 1:**
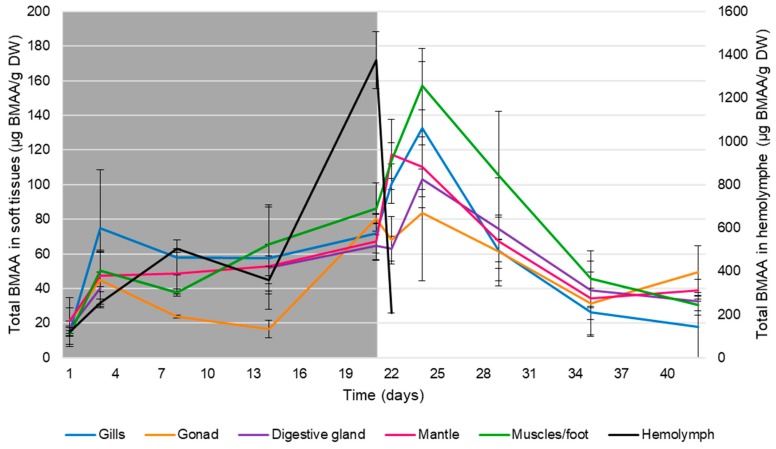
Mean ± SEM of total β-methylamino-L-alanine (BMAA) concentrations in gills (blue), gonad (orange), digestive gland (purple), mantle (pink), muscles and foot (green) and hemolymph (black) of *D. polymorpha* during the exposure (from day 1 to 21: grey area) and the depuration (from day 22 to 42: white area). Regarding the hemolymph, technical difficulties prevented the quantification of total BMAA in samples from day 25 on, although BMAA was detected at each sampling day of the depuration period.

Total BMAA was found in all tissues from the first day of exposure to the last day of depuration (Figure 1). During the exposure, total BMAA concentration in the hemolymph were ranging from 120.7 ± 18.7 µg BMAA/g DW at day 1 to 1373.73 ± 131.94 µg BMAA/g DW at day 22 (first day of depuration). During the depuration, BMAA was detected in the hemolymph up to day 42 (after 21 days in clear water), but technical difficulties prevented the quantification of total BMAA in samples. Indeed, during the depuration, a signal was observed but the absence of signal from the internal standard prevented us from quantifying the presence of BMAA during this period. Overall, during the exposure, total BMAA concentration in the hemolymph was significantly higher than in other tissues (Mann–Whitney test, *p* < 0.001). In soft tissues (gills, mantle, digestive gland, gonad and muscles/foot), no organ appeared to stand out from the others in terms of BMAA concentration during the exposure or the depuration, as no significant differences in total BMAA concentration were observed between tissues during both periods (Kruskal–Wallis test, *p* > 0.05). During exposure, total BMAA concentrations ranged from 13.8 ± 7.4 µg BMAA/g DW (muscles/foot, day 1) to 86.1 ± 15.0 µg BMAA/g DW (muscles/foot, day 21). The overall maximum concentrations of total BMAA in soft tissues were observed at the beginning of the depuration, i.e., at experimental day 22 for the mantle (up to 117.5 ± 20.3 µg BMAA/g DW) and at experimental day 24 for other soft tissues (up to 157.7 ± 24.0 µg BMAA/g DW). The elimination of total BMAA during the depuration was partial as the toxin was still found in tissues after 21 days in clean water, at concentrations up to 49.4 ± 15.3 µg BMAA/g DW. The calculation of the percentage of elimination of total BMAA between the day of maximum accumulation (day 22 for the mantle; day 24 for gills, gonad, digestive gland and muscles/foot) and the last day of depuration (day 42 of the experiment) showed an elimination of 87% of the total BMAA content in gills and of 41% in the gonad. This decrease was significant (Mann–Whitney test, *p* < 0.05) in the digestive gland (−9%), the mantle (−67%) and muscles/foot (−81%). 

**Figure 2:**
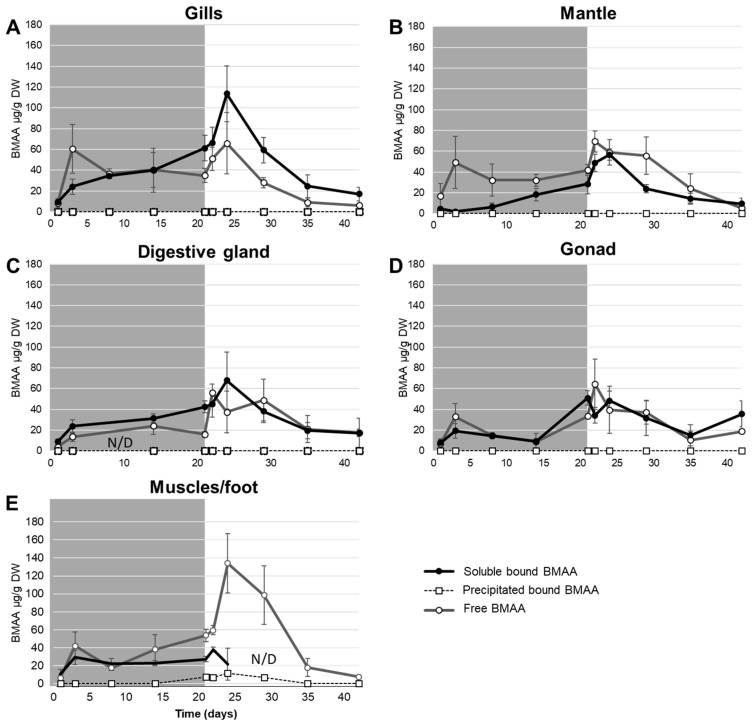
Mean ± SEM concentrations of soluble bound BMAA (●, black lines), precipitated bound BMAA (□, grey lines) and free BMAA (○, dotted lines) in gills (**A**), the mantle (**B**), the digestive gland (**C**), the gonad (**D**) and muscles/foot (**E**) of *D. polymorpha*, during the exposure (from day 1 to 21: grey area) and the depuration (from day 22 to 42: white area). Missing data are indicated as N/D.

Free, soluble bound and precipitated bound BMAA were quantified in each soft tissue along the experiment (Figure 2). No quantification of free BMAA and soluble bound nor precipitated bound BMAA was performed in the hemolymph because of a lack of biological materials. In addition, missing data occurred in the digestive gland (day 8) for all fractions and in muscles/foot (days 29 and 35) only for the soluble bound fraction. Free and soluble bound BMAA were detected in all tissues from the first day of exposure to the last day of depuration (day 42 of the experiment). However, precipitated bound BMAA was only detected in muscles and foot, and only from the last day of exposure (at day 21 of experiment 7.67 ± 1.28 µg BMAA/g DW) to the eighth day of depuration (at day 29 of experiment 6.91 ± 2.44 µg BMAA/g DW).

Except in the mantle during the exposure where free BMAA concentration was significantly higher compared to soluble bound BMAA (Mann–Whitney test, *p* < 0.01), representing overall 62.8 ± 0.2% of total BMAA in this tissue, there were no significant differences between those two fractions in other soft tissues during the entire experiment (Mann–Whitney test, *p* > 0.05). In gills, gonad, muscles and foot, the main quantified fraction varied among tissues (Figure 2) and over time and was alternatively free BMAA or soluble bound BMAA without predominance. However, even if not statistically significantly different, during the depuration, free BMAA concentration were higher than the soluble bound BMAA concentration except in the gonad and gills.

**Figure 3:**
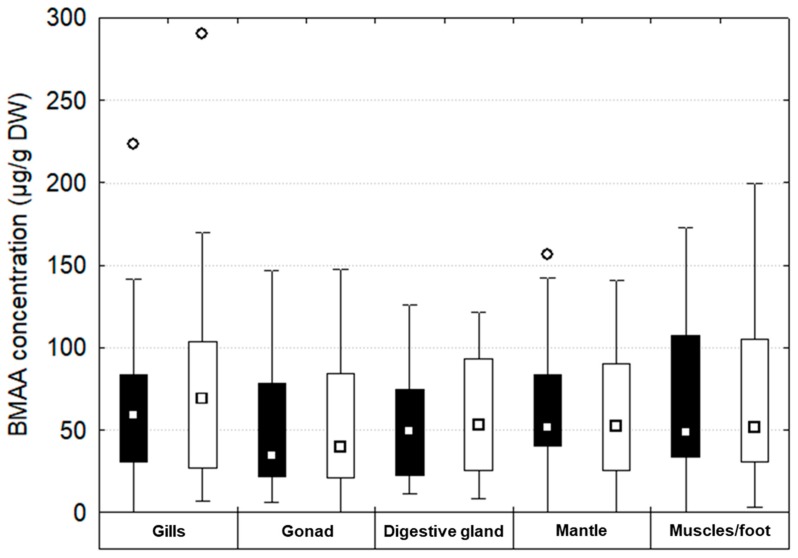
Total BMAA concentration (black boxes) and the sum of free, soluble bound and precipitated bound BMAA (white boxes) in gills, gonad, digestive gland, mantle and muscles/foot of *D. polymorpha* during the entire experiment. The boxplots indicate the first and third quartile of the observations, the whiskers indicate minimum and maximum values, and the median is indicated by a square (n between 27 and 30/condition/tissue).

In soft tissues, total BMAA concentrations were correlated (Pearson’s r = 0.91; *p* < 0.001) with the one evaluated as the sum of free, soluble bound and precipitated bound BMAA in the same samples (Figure 3). No significant differences were observed between those two amounts of total BMAA (calculated or quantified) (Mann–Whitney test, *p* > 0.05).

## 3. Discussion

Here, *D. polymorpha* was exposed to dissolved BMAA for 21 days, followed by 21 days of depuration. The objectives consisted in analyzing the BMAA distribution between the different organs of *D. polymorpha* and the kinetics of the different BMAA fractions (total, free, soluble bound and precipitated bound BMAA) in tissues. We collected bivalves’ hemolymph, which is composed of dissolved proteins and hemocytes. Those circulating cells are primarily in charge of the immune defense and pathogens elimination [38]. They are also known to be implied in various physiological processes, including digestion, tissue repair, shell production, and excretion [38,39,40]. We also collected (i) the mantle, which envelops the hemolymph and inner organs [28]; (ii) gills which are involved in the respiration and the feeding activity as they are implied in food particle catchment and transport to the mouth [41]; (iii) the digestive gland, a tissue were the endocellular digestion occurs; (iv) the gonad and (v) muscles and the foot, a muscular organ where the byssal threads, used for the anchoring to the substrate, are produced.

### 3.1. Organ Distribution of BMAA in D. polymorpha Over Time of Exposure and Depuration

Because *D. polymorpha* is a filter-feeder organism, dissolved BMAA may have been taken-up by mussels through: (i) gills by direct diffusion, and to a lesser extent, (ii) the mantle pathway, and (iii) the digestive tract. As a small hydrophilic amino acid (118.1 Da), BMAA may have been taken up through gills and the mantle, following the same distribution pathway as other dissolved contaminants and amino acids. This is supported by the delay between animals feeding and BMAA addition in the experimental medium, preventing BMAA adsorption on food particles. Small dissolved compounds like metals can directly cross gills and reach the hemolymph and then be distributed to other tissues [42]. The same observation was made with amino acids in *Mytilus californianus* [43]. The transport of amino acids from the medium to tissues through gills can be fast, as 63% of aspartate and 84% of serine were removed from the medium by one mussel during a single passage of water through the mantle cavity [44]. In *Crassostrea gigas*, the neurotransmitter glutamate was found in hemolymph but also in gills, mantle, and muscles after a two-hour exposure to 5.0 µM, suggesting the existence of carriers with broad specificities in theses organs [45]. In the presence of bicarbonate at physiological concentrations, BMAA forms carbamate adducts which are analogue to glutamate, and may therefore be taken-up following the same pathway [46]. BMAA may also have been taken-up through the mantle to a lesser extent. Indeed, the epithelium of this tissue is a unicellular layer, directly exposed to the surrounding medium, which can be crossed by dissolved elements present in the ambient water by active or passive transports mechanisms [27]. Also, BMAA might have been adsorbed on food particles and therefore taken up through the digestive tract. Then, once in the digestive gland, BMAA may have been distributed to other tissues through the hemolymph. A similar mechanism has been suggested for the snail *Lymnaea stagnalis* during exposure to dissolved microcystins (MC) and MC-producing cyanobacteria after MC was detected in snails ‘spermatozoids and oocytes [47]. 

Thus, gills and, to a lesser extent, the mantle and the digestive tract may represent the BMAA entrance route within *D. polymorpha*. The neurotoxin may distribute through the hemolymph to muscles, gonad and digestive gland tissues in which it accumulated without any specific target organ, as demonstrated by our data. BMAA accumulated in all soft tissues from the first day of exposure, which implies that it circulated within a day throughout mussels’ circulatory system. BMAA transfer from the hemolymph to soft tissues can be fast as, after being injected, inulin was mixed completely in the hemolymph within an hour [48]. 

**Figure 4:**
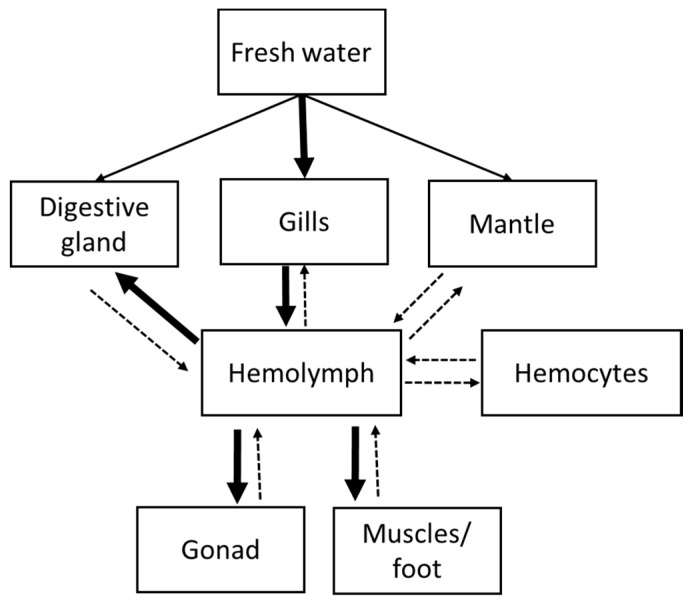
Diagram representation of hypothetical pathway of dissolved BMAA within *D. polymorpha*. Thick arrows indicate major pathways, whereas minor transport is shown by thin arrows. Other hypothetical pathways are indicated in dotted lines. Figure adapted from [50].

Total BMAA concentration in the hemolymph was higher (ranging from 120.7 ± 37.2 µg/g DW to 1373.7 ± 131.9 µg/g DW) compared to soft tissues (ranging from 13.8 ± 7.4 µg/g DW in gills to 157.1 ± 13.9 µg/g DW in muscles/foot). Mussel hemolymph is composed of circulating cells (hemocytes) and plasma, composed of dissolved proteins. It is plausible that those two constituents were involved in the transport of BMAA in tissues of *D. polymorpha*. This is supported by the exposure of the bivalve *Mercenaria mercenaria* to 100 ng/L of cadmium where Cd concentration in the hemolymph was also higher compared to soft tissues and disparities existed between Cd concentration in hemocytes and Cd concentration in the plasma. Indeed, after 10 h, less than 10% of Cd was found in hemocytes, around 35% in soft tissues and roughly 60% was found in the plasma [49]. The transfer of BMAA to tissues through the open-circulatory system might explain the observed increasing concentration of total BMAA in tissues during the exposure and from the first to the third day of depuration. Indeed, total BMAA concentrations increased by 4.0% (gonad) to 84.9% (gills) between day 21 and 24, three days after mussels were placed in clean water. After the third day of depuration (day 24), the total BMAA content decreased in all organs, demonstrating a detoxification, albeit partial as concentration of total BMAA at the 21st day of depuration represented 41% (gonad) to 87% (gills) of the maximum concentration of total BMAA observed at day 22 (mantle) or 24 (gills, gonad, digestive gland and muscles/foot). It can be hypothesized that BMAA transport from hemolymph to tissues was not one-way, and that small proportion of BMAA could be transported from tissues to the hemolymph to be excreted by an unknown route. Thus, a hypothetical pathway of dissolved BMAA within *D. polymorpha* can be proposed and would require further investigation (Figure 4). Moreover, further analyses of BMAA concentration in the hemolymph during the depuration are required to complete this study and have a better understanding of the role of this compartment in BMAA elimination.

### 3.2. BMAA Accumulation Fractions in Tissues of D. polymorpha

Soluble bound and free BMAA represented the main forms of accumulation in *D. polymorpha*. Precipitated bound BMAA was only quantified in muscles/foot during a small period covering 9 days. No differences existed between each total BMAA quantification and the sum of corresponding free-soluble bound and precipitated bound BMAA, demonstrating that all possible fractions were included. Previous exposure of *D. polymorpha* to 100 µg of radiolabelled BMAA/L showed a BMAA accumulation in both free and the precipitated bound fractions [28]. In this study, soluble bound ^5+^BMAA was not analysed and discrepancies between the estimated total amount of BMAA removed from the medium by *D. polymorpha* and the total amount of BMAA detected in mussels were observed [28]. 

More analyses are needed to understand the process to which BMAA binds itself to molecules of low or high molecular weight and to identify the molecules involved in the association with BMAA. 

In the present study, after 21 days in clean water, BMAA was mostly in the soluble bound fraction except in muscles/foot where free BMAA was predominant. Therefore, the soluble bound fraction might represent the long-term accumulation form of BMAA within mussels. These results are consistent with studies carried out with *Mytilus edulis*, seafood and daphnids [29,32]. These data also comfort the hypothesis that the omission of the measurement of soluble bound BMAA could potentially induce an underestimation of the total BMAA concentration in a sample, while the sole analysis of this fraction would not lead to a strong underestimation of the total BMAA concentration in the same sample [13,29].

### 3.3. BMAA Kinetics of Accumulation and Detoxification Within Tissues

Total BMAA concentrations varied during the study: for the first three days of exposure, BMAA concentration within each tissue increased rapidly by 2.1 (gonad) to 5 times (gills). This was also observed during an exposure of *D. polymorpha* to 100 µg ^5+^BMAA/L for 24 and 48 h [28]. In our study, a stagnation of BMAA accumulation rate was observed from day 3 to 14 in the hemolymph, digestive gland, mantle and muscles/foot. Moreover, BMAA concentration at day 14 was 1.3 times higher compared to day 3. In the same period, BMAA concentration decreased in gonads and gills by 0.6 times, when BMAA was still present in the medium. This lower accumulation rate may have occurred because of a potential metabolization or a detoxification of BMAA. However, as BMAA is a hydrophilic compound, the biotransformation of this amino acid by the enzyme glutathione-S-transferase (GST) is not expected [28], despite the demonstrated impact of BMAA on the GST activity. Indeed, BMAA either promoted inactivation of GST activity with purified enzymes [51], or activated it, as observed in *D. polymorpha* exposed 24 h to 500 µg BMAA/L [52]. BMAA was detected mainly in the soluble bound fraction and sporadically in the precipitated bound fraction, a conjugation of the potent BMAA adduct with glutathione catalysed by GST to be further eliminated could be hypothesized. Free BMAA might be released during this biotransformation, favoring the presence of free BMAA in tissues. During the depuration, soluble bound and free BMAA represented the main forms of BMAA accumulation in soft tissues and their elimination was partial. The metabolization and potential detoxification pathways of BMAA remain to be investigated.

In addition, further studies should be carried out to study the evolution of free, soluble bound and precipitated bound BMAA during the depuration in the hemolymph, in order to better understand the transport of these fractions over time. This analysis was not possible here because the quantity of hemolymph we collected from two individual mussels per replicate only allowed analyzing total BMAA concentration after being freeze-dried. We estimate that such an analysis would require to pool hemolymph from more than ten animals.

### 3.4. Use of Bivalves to Monitor Environmental BMAA: Pertinent Fractions and Tissues

To monitor environmental toxins such as microcystins (MC), invertebrates’ digestive glands or vertebrates’ livers are often sampled in relation with the higher density of specific transporters in those tissues compared to others [53,54,55]. This organotropism occurs regardless of the intoxication pathway, i.e., ingestion of MC-producing organisms or exposure to dissolved MC [47,56]. This study demonstrated that unlike MC, BMAA was diffused throughout all soft tissues, without a specific target organ and that omitting a tissue may underestimate the overall level of contamination of the individual. As the highest BMAA concentrations were observed in the hemolymph during exposure, the analysis of this tissue could potentially reveal the presence of BMAA-producing organisms in the water. However, hemolymph sampling is delicate. Quantification of BMAA in the entire animal may therefore be more appropriate to reveal environmental concentration levels. Depending on the size of the organism, only one organ may be selected, but considering that the BMAA content will only represent a small proportion of the BMAA accumulated in the entire body. Moreover, this study also showed that no BMAA accumulation fraction should be omitted when analysing BMAA concentration in soft tissues to prevent an underestimation of BMAA concentration in whole individuals. Therefore, the total BMAA fraction is to the most relevant fraction to analyse, when studying BMAA concentration in freshwater bivalves.

## 4. Conclusions

The study of the BMAA organ distribution within *D. polymorpha* and of the dynamics of its different accumulation fractions showed that BMAA was found in all tissues from the first day of exposure to the 21st day of depuration, without any specific target organ. Main entrance pathway may be through the mantle and gills, and then BMAA was transferred to other tissues via the hemolymph. However, the ingestion of BMAA adsorbed on particles or via water ingestion remained possible, but less significant. In soft tissues, BMAA was mainly free or in the soluble bound fraction with fluctuations between these two fractions over time, and BMAA was detected in the precipitated bound fraction only in the muscle during few days. Because the soluble-bound fraction was the main fraction in soft tissues during the 21-day depuration, except in the mantle and muscles/foot, this fraction may represent the long-term accumulation form of BMAA in bivalves. In the context of the evaluation of freshwater contamination by BMAA using mussels as bioindicators, this study highlighted the importance of sampling whole individuals when possible and to analyse the concentration of total BMAA to prevent the underestimation of BMAA contamination levels.

## 5. Materials and Methods 

### 5.1. Mussels Sampling and Acclimation

Individuals of *D. polymorpha* were collected in March 2017 at the Lac-du-Der-Chantecoq (48°36′07.7″ N; 4°44′37.0″ E) around 5 m depth. Then mussels measuring 2 ± 0.3 cm were randomly dispatched in groups of 275 mussels in two 3 L aerated tanks containing half of water from the sampling site and half of Cristalline^®^ source water (Saint Yorre, France). Mussels were kept at 16 ± 2 °C with a 12 h:12 h light: dark cycle. After three days, the two-week acclimation started when the water was removed and replaced with 100% Cristalline^®^ source water. Individuals of *D. polymorpha* were fed twice a week with 2 × 10^6^ cells/mussel/day of a 50:50 mix of *Chlorella vulgaris* and *Scenedesmus obliquus* obtained from Greensea (Mèze, France). Algal density was measured with a light microscope (Primovert, Zeiss, Oberkochen, Germany) and KOVA^®^ slides (Kova slide, VWR, Fontenay-sous-Bois, France). Briefly, cultures were gently shaken, and after homogenization, three replicates of 1 mL were sampled, diluted ten or twenty times. Cells were numbered twice per replicate by placing 10 µL in chambers. The water was renewed every three days.

### 5.2. Exposure

#### 5.2.1. Experimental Setting

**Figure 5:**
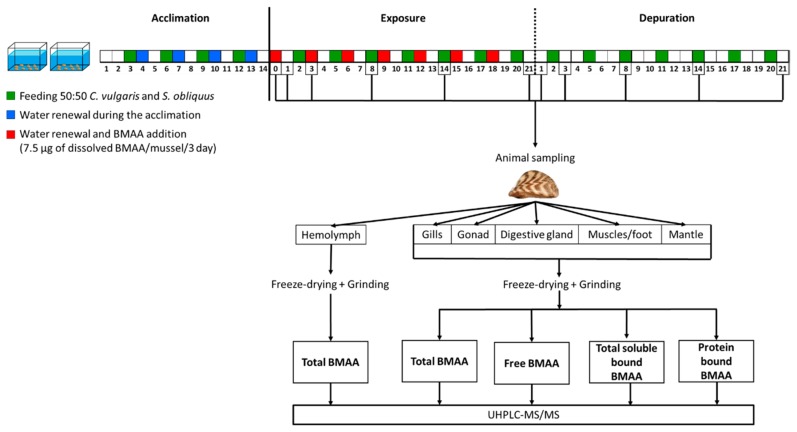
Graphical representation of the experimental design.

Before the experiment, three control mussels per tank were randomly collected and dissected to quantify the BMAA within tissues. Then, *D. polymorpha* were exposed 21 days to dissolved BMAA (L-BMAA hydrochloride B-107, Sigma–Aldrich^®^, Saint-Louis, MO, USA). The experiment was semi-static as the water was renewed every three days with nominal concentration of 7.5 µg dissolved BMAA/mussel/3 days (Figure 5). The study was carried out in duplicates, with two 3L-tanks each containing 275 mussels. Mussels were fed as described during the acclimation (2 × 10^6^ cells/mussel of a 50:50 mix of *C. vulgaris* and *S. obliquus*), every three days and not long before water changing, to minimize BMAA adsorption on food particles. After the 21 days exposure period, mussels were transferred in new tanks containing only clean water, for a 21 day depuration period and fed as described during the exposure.

#### 5.2.2. Tissue Sampling

Three mussels per tanks were randomly collected and placed for 24 h in clear water before being sacrificed. Mussel were collected during the exposure phase on days 0 (control), 1, 3, 8, 14 and 21 and during the depuration phase on days 22, 24, 29, 35 and 42. For each mussel, the hemolymph was withdrawn from the posterior adductor muscle using 300 µL-Myjector^®^ (Terumo, Tokyo, Japan) syringes, and transferred into Eppendorf^®^ tubes (Eppendorf, Hamburg, Germany). Then gills, the digestive gland, the gonad, the mantle and muscles/foot were separated, transferred into Eppendorf^®^ tubes and pooled to form three pools of two individuals, each coming from different tanks, per sampling time. Samples were put in liquid nitrogen and stored at −80 °C. After being freeze-dried, tissues were grinded with a Mixer Mill MM400 (Retsch, Haan, Germany) using 4 beads and 4 min of beating at 30 Hz.

### 5.3. Extraction

Extraction of total, free, soluble bound and precipitated bound BMAA was performed as described in the literature [31].

#### 5.3.1. Total BMAA

Tissues were weighted and 1 mg of freeze-dried tissues were spiked with 40 µl D_3_BMAA in 20 mmol/L HCl, as an internal standard, then dried under a vacuum. After the addition of 30 µL 6 M HCl, tissues were hydrolysed at 0.7 mbar for 20 h at 105 °C in an Eldex^®^ hydrolysis workstation (Eldex, Napa, CA, USA). After being dried under vacuum, they were resuspended twice in 500 µL 67:33:0.1 ACN:water:formic acid mix and transferred into spin filter tubes and centrifuged before analysis.

#### 5.3.2. Free and Soluble Bound BMAA

As described in the literature [31], 12.5 mg of freeze-dried tissues were spiked with 40 µL D_3_BMAA in 20 mmol/L HCl, dried under a vacuum and extracted twice with 600 µL 0.1 M trichloroacetic acid (TCA). After centrifugation with spin filters, the sample was split into two fractions: one part of the supernatant was used for the extraction of free BMAA. The filtrate was dried with a Thermo Savant SPD121P Speed Vac (Thermo Scientific, Basingstoke, UK), and resuspended with 500 µL 67:33:0.1 ACN:water:formic acid mixed before analysis. The other part was used for the extraction of the total amount of BMAA that is soluble in TCA (“total soluble BMAA”) corresponding to free and soluble bound BMAA. The filtrate was freeze-dried, hydrolysed with 30 µL 6 M HCl as described in Section 5.3.1 and then resuspended twice with 250 µL 67:33:0.1 ACN:water:formic acid mix before analysis. The concentration of soluble bound BMAA was calculated by subtracting the concentration of “free BMAA” from the concentration of “total soluble BMAA”.

#### 5.3.3. Precipitated Bound BMAA

As described in the literature [31], 1 mg of freeze-dried tissues was extracted twice with 150 µL of 0.1 M TCA. The supernatant was discarded, and the pellet was processed as described for total BMAA in Section 5.3.1.

### 5.4. UHPLC-MS/MS Analysis

Due to different equipment availabilities, the samples were analysed on two different LC-MS/MS systems.

The hemolymph samples were analysed as described in [57] on the same type of UHPLC-MS/MS system (Waters Acquity UHPLC coupled to an Xevo TQS with ESI interface) but using a 150 mm column instead of a 100 mm column with a 5 mm precolumn. BMAA was separated from DAB and AEG; the retention time of BMAA and D_3_BMAA was 9.4 min, the retention time of DAB was 10.8 min, and the retention time of AEG was 11.8 min. For BMAA, the ion ratios were as follows: *m*/*z* 102:88, 27%; *m*/*z* 102:76, 27%; *m*/*z* 102:73, 20%; and *m*/*z* 102:44, 52%. For D_3_BMAA, the ratios were *m*/*z* 105:88, 31%; and *m*/*z* 105:76, 23%. Retention times and ion ratios were comparable to those reported in [57]. 

All other samples were analysed on the UHPLC-MS/MS system 1290 Infinity II connected with a 6490C triple quadrupole MS (Agilent, Santa Clara, CA, USA). This method was slightly modified from the underivatised BMAA analysis described in the literature [30]. The compounds were separated on a HILIC column (ZIC^®^HILIC, 150 × 2.1 mm, 5 µm, 200 Å, Merck, Darmstadt, Germany) set at 40 °C, 5 µL was injected. The mobile phase consisted of acetonitril with 0.1% formic acid (A) and MilliQ-water with 0.1% formic acid (B). The initial conditions were 5% B for 2 min, followed by a gradient from 2 to 4 min to 35% B, from 4 to 8 min to 45% B and hold till 16 min at 45% B. Between 16 and 17 min, B was decreased to 5%, and this was held for another 5 min. The mass spectrometer was used in the positive mode with a gas flow of 12 mL/min, a source temperature of 230 °C a nebulizer pressure of 40 psi, a sheath gas temperature of 200 °C, sheath gas flow of 12 l/min, capillary voltage of 2.5 kV. The compounds were analysed in Multi Reaction Mode (MRM) using nitrogen as collision gas. BMAA was monitored by the transitions *m*/*z* 119.1 > 76.2, 119.1 > 88.1 and 119.1 > 102.1 using a collision energy of 9, 9 and 5 V and a fragmentor voltage of 73 V. Transitions for DAB were *m*/*z* 119.1 > 101.1 and 119.1 > 74.2 using a collision energy of 5 and 13 V and a fragmentor voltage of 68 V. D_3_BMAA was monitored by the transitions *m*/*z* 122.1 > 76.2, 122.1 > 88.1 and 122.1 > 105.1 using a collision energy of 9, 9 and 5 V and a fragmentor voltage of 75 V. For data-acquisition and analysis Masshunter B 08.02 (Agilent, Santa Clara, CA, USA).

For both methods, a 20% relative deviation from the average ion ratios in the standards was allowed in the samples. The BMAA retention time was verified by D_3_BMAA retention time. BMAA was quantified against an external calibration curve and the concentrations in each sample were corrected for the signal intensity of the internal standard. DAB and AEG were not quantified in this study, but only included in the analysis to ensure that there was no co-elution with BMAA.

### 5.5. Statistics

Statistical analyses were performed with Statistica (Version 8.0.360.0, Statsoft, Tulsa, USA, 2007). Normality was checked for with a Shapiro–Wilk test, and the homogeneity of variances was studied with a Levene’s test, as both tests had a *p*-value below 0.01, non-parametric tests were used. Therefore, the comparison of multiple independent samples was done with Kruskal–Wallis tests and comparisons between two datasets were made with Mann–Whitney tests.
